# When Does Status Turn Into Proactive Helping Behavior? The Moderating Role of Cooperative/Competitive Behavior Intention

**DOI:** 10.3389/fpsyg.2019.02702

**Published:** 2019-12-03

**Authors:** Chuanjun Deng, Shudi Liao, Zhiqiang Liu

**Affiliations:** ^1^Business School, Henan University, Kaifeng, China; ^2^Business School, Hubei University, Wuhan, China; ^3^School of Management, Huazhong University of Science and Technology, Wuhan, China

**Keywords:** self-perceived status, proactive helping behavior, cooperative behavior intention, competitive behavior intention, status competition

## Abstract

Self-perceived status is considered an important antecedent of an employee’s extra-role behavior. However, the relationship between self-perceived status and “proactive helping” has been neglected in previous human resource management (HRM) research. Data were collected from 214 employees and their supervisors in two waves of dyads. The results of hierarchical regression analysis indicated that self-perceived status is positively related to proactive helping behavior, while cooperative and competitive behavior intentions were both found to have a moderating effect on the relationship between self-perceived status and proactive helping behavior. Specifically, the relationship between self-perceived status and proactive helping behavior became stronger as the cooperative behavior intention increased, but weakened as competitive behavior intention increased. These conclusions indicate that organizations should guide employees to enhance their cooperative behavior intention or decrease their competitive behavior intention, which may increase their willingness to proactively help others. The study’s theoretical and practical contributions and future research are discussed.

## Introduction

The structure of proactive helping behavior was first examined by Spitzmuller and Van Dyne (2013), and has since become a topic of debate in academia. They defined proactive helping behavior as an employee offers help to other colleagues without asking for help. Proactive helping behavior is conducive to the improved job performance of an employee’s teammates and enhances team efficiency. This behavior both conforms to the expectations of leaders and colleagues and fits with social norms. However, employees’ proactive helping behavior can be initiated by the individual’s self-interest and based on personal needs (Spitzmuller and Van Dyne, 2013; Bamberger and Belogolovsky, 2017). It may, for example, be beneficial to the individual’s impression management and increasing their influence (Flynn et al., 2006; Willer, 2009) as well as encouraging more cooperation and help from others. Willer (2009) and Simpson et al. (2012) also believed that status may drive generosity because high-status members will be more likely than others to make initial contributions to the workgroup. From above we can infer that there is a certain relationship between self-perceived status and employee’s proactive helping behavior. However, the antecedents of proactive helping behavior have been examined and limited to factors such as transformational leadership (Zhu and Akhtar, 2014), authentic leadership (Hirst et al., 2016), and so forth in the previous articles. No research to date has explored and empirically tested the formation mechanism of employees’ proactive helping behaviors from the perspective of status competition. The theory of status competition (Loch et al., 2000; Gould, 2002; Willer, 2009; Simpson et al., 2012) suggests that the higher the self-perceived status, the more attention will be devoted to image maintenance, and thus the willingness to spend more energy and resources on attaining the goal will increase. High-status members are more eagle to control the outcome by proactive helping behavior (Anderson et al., 2015; Hays and Bendersky, 2015). Therefore, they will pay more attention to improving their image by developing a positive reputation with their colleagues (Flynn et al., 2006). It can be inferred that self-perceived status is likely to be an important antecedent of employees’ proactive helping behavior.

The individual pursuit of career development may choose both cooperative and competitive behaviors (Chang et al., 2017; Arslan, 2018). These different behavior intention types will lead to significant differences in employees’ attention and behavior selections. When an employee engages in cooperative behavior, they will focus on sharing valuable resources with others (Chang et al., 2017), and will believe that the status achievement of others will help them (Chen et al., 2006). However, the employees engaging in competitive behavior to pursuit career success will mainly consider how to protect their benefit by blocking the dissemination of valuable information to others (Gibson et al., 2018) and intentionally undermining others (Garcia et al., 2013), because they believe that when others are winners, they will be less likely to succeed (Chen et al., 2006). Thus, we can assume that the moderated effects of cooperative behavior intention on the relationship between self-perceived status and proactive helping will be very different from the effects of competitive behavior intention. And these types of behavior intention have not been adequately examined in previous research. Therefore, we integrated these two types of intention into the theoretical framework that can deepen us to understanding the mechanisms of self-perceived status influence on individual proactive helping behavior (Hays and Bendersky, 2015; Chang et al., 2017).

This study makes three main theoretical contributions. First, by drawing on the theory of status competition (Loch et al., 2000; Willer, 2009; Simpson et al., 2012) we extend our understanding of the effects of employee self-perceived status on proactive helping, and add to the increasing body of literature that explores the antecedents of employee proactive helping. Second, based on the theory of status competition, we distinguish the moderating effect of competitive behavior intention from that of cooperative behavior intention, and thus provide insights into how status-striving behavior motives can improve or undermine employees’ proactive helping behavior. Third, this study is the first to analyze and empirically test the effects of cooperative versus competitive behavior intention at the individual level. As previous research has mainly explored the effects of these two types of behavior intentions at the group level (Chang et al., 2017), this study enriches the literature concerning individuals’ behavioral intentions.

## Hypotheses

### Self-Perceived Status and Proactive Helping

In this study, it is proposed that there is a positive correlation between self-perceived status and proactive helping behavior, based on the following theoretical evidence. First, from the perspective of status maintenance, the higher individuals’ self-perceived status, the more they will want to invest in maintaining their image. Offering proactive helping behavior can enhance an individual’s image and bring additional benefits, such as others’ increased willingness to collaborate and help (Nowak, 2006), positive feedback and delegation from leaders (Bolino, 1999), and personal development and career success (Clary and Snyder, 1999). Therefore, the higher the self-perceived status, the more obvious the willingness to help others.

Second, from the perspective of status advantages, higher self-perceived status individuals believe that their proactive helping behaviors are more likely to be accepted by others than lower self-perceived status employees. High-status employees have been found to obtain more approval and support from others than low-status employees (Tortoriello et al., 2011), and they often take the initiative to help others and consequently achieve high-quality cooperation (Brandts et al., 2015), thus further consolidating their status. For instance, other studies have found that employees with higher self-perceived status are more willing to be generous to others than their peers so they can win their trust (Lount and Pettit, 2012).

Third, those with higher self-perceived status may perceive their value as higher because individual status is conferred by other teammates, and thus motivates them to develop proactive helping behaviors. The external social expectations of high-status team members are significantly higher than those of low-status members (Anderson et al., 2012b). To reciprocate the appreciation and respect of their teammates, higher self-perceived status members must actively convey messages of warmth and caring to others (Flynn, 2003; Fragale et al., 2011; Torelli et al., 2014). Based on the above analysis, we propose the following hypothesis:

*Hypothesis 1:* Self-perceived status is positively related to employee proactive helping behavior.

### The Moderating Effects of Behavior Intentions

Individual behavior intentions can be cooperative or competitive. Cooperative behavior intention is defined as the pursuit of status through cooperation with colleagues (Chang et al., 2017), while competitive behavior intention is defined as the pursuit of status through negative competition with others (Chang et al., 2017). The goal of status competition can be achieved through cooperative or competitive behavior. Some may choose to achieve status goals by sacrificing their own interests for those of their work team or teammates (Willer, 2009; Chang et al., 2017), while others may choose to achieve these goals by reducing cooperation, information blocking, etc. (Metiu, 2006; Ridgeway, 2014). Further distinguishing the influences of these opposing types of behavior intention can effectively deepen our understanding of why the proactive helping behaviors of some employees significantly improve, while those of others significantly decline.

Employees who have cooperative behavior intentions believe that as one member moves toward attaining their status goal, others also move toward achieving their goals (Chen et al., 2006). They believe that if others achieve their goals it will help them, creating a win–win situation, so their proactive helping intention will be increased. Studies have found that when individuals’ cooperative behavior intention is high (rather than low), they will choose to form alliances with others to expand their social networks (Thye, 2000), and thus they will offer more proactive help to others (Halabi et al., 2008). Unlike those with low cooperative behavior intention, employees with high cooperative behavior intention take the initiative in collaborating with others, and this willingness to collaborate will help them improve their proactive helping behaviors (Johnson and Johnson, 2005; Kistruck et al., 2016). A high cooperative behavior orientation will significantly enhance the attitude of wanting to actively help others, and proactive helping behavior will then obviously increase (Bock et al., 2005). The proactive helping behaviors of high-status members are more acceptable to the group members on account that they are considered more competent (Flynn and Amanatullah, 2012). And they can use proactive helping to foster efficient coordination (Brandts et al., 2015). Therefore, with the influences of high cooperative behavior intention, high self-perceived status members will increase their proactive helping more obviously than others. We thus propose the following hypothesis:

*Hypothesis 2:* Cooperative behavior intention strengthens the relationship between self-perceived status and proactive helping such that the relationship is more positive when cooperative behavior intention is high rather than low.

If their behavior intention is competitive, employees believe that their goal attainment realization will make others less likely to achieve their status goals (Chen et al., 2006). When others achieve high performance and influence, they will be less likely to succeed. These employees want to be the winners and want others to be the losers. Therefore, with the influences of competitive behavior intention, higher self-perceived status employees are more likely to decrease their proactive helping behavior than those lower self-perceived status members. Specifically, when their competitive behavior intention is high (rather than low), they will focus on how to improve their competitiveness while weakening that of their peers, which will lead them to intentionally hinder the progress of others (Garcia et al., 2013). For example, they may stealthily block the dissemination of valued information, as suggested by the theory of closure (Ridgeway, 2014), intentionally attempt to hide knowledge that others critically need, and implicitly resist cooperation with colleagues (Metiu, 2006).

Furthermore, under the influences of competitive behavior intention, high-status employees may also believe that status-contrasting has a zero-sum outcome, and are thus likely to engage in status conflict with or behave antagonistically toward peers because they regard others as rivals. Their willingness to actively help others is thus significantly lower (Garcia et al., 2013). Studies have shown that when status conflicts or relationship conflicts occur, high-status members’ willingness to actively help others is decreased more obviously than their peers (Pettit et al., 2010; Bendersky and Hays, 2012). A competitive behavior orientation has also been found to lead to a zero-sum outcome in status competition among individuals, and to increased bystander engagement (Bendersky and Hays, 2012).

Additionally, competitive behavior intention can encourage social comparison to the high-status members more obviously than the low-status, thus producing feelings of envy and perceptions of injustice toward those who appear better, and hostility to those who are equal in terms of competitiveness (Obloj and Zenger, 2017; Gerber et al., 2018). Those who appear inferior in terms of competitiveness may also be targets for status and information closure (Bidwell, 2013; Ridgeway, 2014). All of these situations will more significantly decrease the willingness of high self-perceived status members to engage in proactive helping than other colleagues. Based on the above analysis, we propose the following hypothesis:

*Hypothesis 3:* Competitive behavior intention moderates the relationship between self-perceived status and proactive helping such that the relationship is less positive when competitive behavior intention is high rather than low.

**FIGURE 1:**
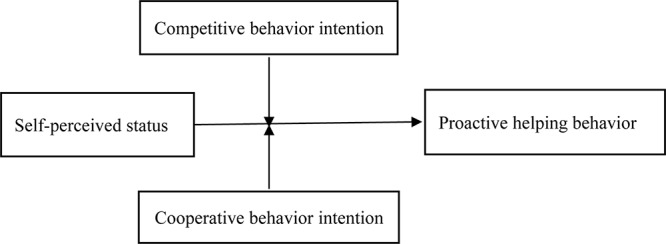
Research framework.

Figure 1 presents the study’s theoretical framework.

## Materials and Methods

### Sample and Procedures

Data were collected over about 6 months from 17 companies and enterprises in China. Most were in the service industry or the financial industry. Only full-time employees of these organizations were invited to take part in the research. Of the 347 employees who participated in the first-wave collection (T1), 271 provided usable data (overall response rate = 78.10%). The supervisors of those employees participated in the second wave (T2) of data collection, and 56 provided valuable data about their subordinates’ proactive helping behaviors. All the members answer the questionnaire independently, and the surveys returned to us in sealed envelopes. Finally, 208 valuable supervisor–subordinate dyadic data pairs were collected. About 50.5% of the employees in the final sample were male, and 49.5% were female. About 37% of those members’ education were bachelor degree and master degree, and about 63% were college degree. And the average tenure was 6.60 years (standard deviation 5.96), with an average age of 29.20 years (standard deviation 5.93).

### Measures

#### Self-Perceived Status (T1)

A three-item scale adopted from Janssen and Gao (2015) was used to capture employees’ self-perceived status. A sample item is “I have a great status in the work team.” The participants responded on a 5-point Likert scale, ranging from 1 (strongly disagree) to 5 (strongly agree). Cronbach’s α was 0.82.

#### Cooperative Behavior Intention (T1)

This was self-reported by the participants. A four-item scale was used to measure their competitive behavior intention (Chang et al., 2017). A sample item is “I want to improve my competitiveness by sharing my expertise with other members actively.” The participants responded on a 5-point Likert scale, ranging from 1 (not at all) to 5 (very much). Cronbach’s α was 0.90.

#### Competitive Behavior Intention (T1)

This was self-reported by the participants. A four-item scale was used to measure their cooperative behavior intention (Chang et al., 2017). A sample item is “If other colleagues in my work team ask me for expertise, I want to keep them from improvement by ostensibly agree to help with.” The participants responded on a 5-point Likert scale, ranging from 1 (not at all likely) to 5 (very likely). Cronbach’s α was 0.87.

#### Proactive Helping Behaviors (T2)

The employees’ proactive helping behaviors were evaluated by their supervisors. A three-item scale developed by Lee et al. (2019) was used to measure the employees’ willingness to proactively help behavior. A sample item is “Without being asked, this employee often actively help their teammates who had task-related problems.” The participants responded on a 5-point Likert scale, ranging from 1 (never) to 5 (always). Cronbach’s α was 0.76.

#### Control Variables (T1)

Based on suggestions made by Janssen and Gao (2015) and Chang et al. (2017), the variables included gender (1 = male, 0 = female), age, education (1 = below Bachelor’s degree and 0 = above Bachelor’s degree), and tenure. We also created two dummy variables to control for three industries (service industry, financial industry, and others).

## Results

The confirmatory factor analysis (CFA) results using AMOS 7.0 confirmed the discriminant validity of self-perceived status, proactive helping behavior, competitive behavior intention, and cooperative behavior intention. The predicted four-factor model (χ^2^/*df* = 1.86, RMSEA = 0.06, GFI = 0.92, IFI = 0.96, and CFI = 0.96) was a better match to the data than the one-, two-, and three-factor models (Table 1), and thus the results supported our treatment of the variables as measuring distinct constructs.

**TABLE 1:** Confirmatory factor analyses.

**Model**	**χ^2^**	***df***	**χ^2^/*df***	**RMSEA**	**GFI**	**IFI**	**CFI**
Four-factor model (SS/CBI/COBI/PHB)	132.03	71	1.86	0.06	0.92	0.96	0.96
Three-factor model (SS/CBI/COBI + PHB)	297.59	74	4.02	0.12	0.83	0.86	0.86
Three-factor model (SS/COBI/CBI + PHB)	300.03	74	4.05	0.12	0.82	0.86	0.86
Three-factor model (SS/PHB/COBI + CBI)	491.98	74	6.65	0.17	0.72	0.73	0.73
Three-factor model (CBI/COBI/SS + PHB)	201.99	74	2.73	0.09	0.88	0.91	0.91
Three-factor model (CBI/PHB/SS + COBI)	376.54	74	5.09	0.14	0.79	0.81	0.81
Three-factor model (PHB/COBI/SS + CBI)	627.83	74	8.48	0.19	0.66	0.65	0.64
Two-factor model (SS/CBI + COBI + PHB)	573.04	76	7.54	0.18	0.69	0.67	0.66
One-factor model (SS + CBI + COBI + PHB)	812.05	77	11.33	0.22	0.62	0.51	0.50

*SS, self-perceived status; CBI, competitive behavior intention; COBI, cooperative behavior intention; and PHB, proactive helping behavior.*

Table 2 reports the means, standard deviations, and correlations of the variables. All of the reliability estimates were acceptable (i.e., α > 0.70).

**TABLE 2:** Means, standard deviation, and correlations (*N* = 208).

**Variables**	***M***	***SD***	**1**	**2**	**3**	**4**	**5**	**6**	**7**	**8**
1. Age	29.20	5.93								
2. Tenure	6.60	5.96	–0.85^∗∗^							
3. Financial industry	0.15	0.36	–0.13	–0.11						
4. Service industry	0.53	0.50	0.09	0.08	−0.46^∗∗^					
5. Proactive helping behavior	3.77	0.81	0.19^∗∗^	0.13	0.04	0.07	(0.76)			
6. Competitive behavior intention	1.69	0.95	–0.03	–0.06	−0.05	−0.05	−0.05	(0.87)		
7. Cooperative behavior intention	4.26	0.78	0.09	0.12	−0.03	0.11	0.06	−0.41^∗∗^	(0.90)	
8. Self-perceived status	2.54	0.99	–0.07	–0.01	−0.07	0.07	0.17^∗^	0.04	0.08	(0.82)

*^∗^*p* < 0.05 and ^∗∗^*p* < 0.01 (two-tailed).*

**FIGURE 2:**
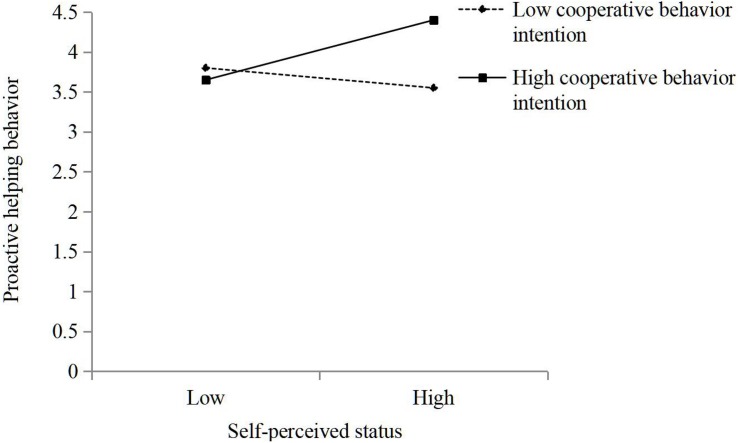
The moderating role of cooperative behavior intention in the relationship between self-perceived status and proactive helping behavior.

**FIGURE 3:**
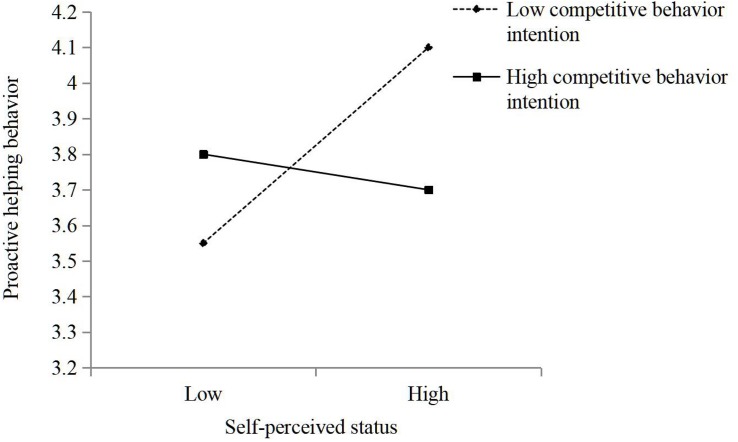
The moderating role of competitive behavior intention in the relationship between self-perceived status and proactive helping behavior.

We first standardized the independent and moderated variables before hierarchical analysis. Hypothesis 1 predicted that self-perceived status is positively connected to proactive helping behavior. The results of the hierarchical multiple regression analysis in Model 2 in Table 3 show that self-perceived status was positively related to employees’ proactive helping behavior (β = 0.16, *p* < 0.01). It indicates that high self-perceived status employees will be more willing to proactive helping than those low-status peers. Therefore, Hypothesis 1 was supported. Hypothesis 2 suggested that cooperative behavior intention moderates the relationship between self-perceived status and proactive helping behavior, such that the connection is stronger when self-perceived status is high rather than low. The results of Model 3 in Table 3 show that the coefficient for the interaction between self-perceived status and employee’s proactive helping behavior was significant and positive (β = 0.22, *p* < 0.001). And Hypothesis 3 predicted that competitive behavior intention moderates the relationship between self-perceived status and employee’s proactive helping behavior such that the connection is weaker when competitive behavior intention is high rather than low. The results of Model 4 in Table 3 show that the coefficient for the moderation between self-perceived status and employee’s proactive helping behaviors was significant and negative (β = −0.20, *p* < 0.01). As a robustness check, we ran our model with both competitive behavior intention and cooperative behavior intention with similar results in Model 5 in Table 3. And the results in Model 5 were plotted for cooperative/competitive behavior intention values corresponding to one standard deviation below and above the mean (Figures 2, 3). To further analyze this moderation effect, a simple slopes test showed that the slope for low cooperative behavior intention was non-significant (γ = −0.16, n.s.), and the slope for high cooperative behavior intention was positive and significant (γ = 0.34, *p* < 0.001). And the similar results showed that the slope for low competitive behavior intention was significant (γ = 0.28, *p* < 0.001), and the slope for high competitive behavior intention was non-significant (γ = −0.05, n.s.). Thus, Hypotheses 2 and 3 were supported.

**TABLE 3:** Results of hierarchical regression analysis (*N* = 208).

**Variables**	**M1**		**M2**		**M3**		**M4**		**M5**	
	**β**	**SE**	**β**	**SE**	**β**	**SE**	**β**	**SE**	**β**	**SE**
Control variables										
(Constant)	2.49^∗∗∗^	0.45	2.36^∗∗∗^	0.44	2.33^∗∗∗^	0.43	2.23^∗∗∗^	0.44	2.25^∗∗∗^	0.43
Sex	–0.01	0.11	–0.03	0.11	–0.05	0.11	–0.00	0.11	–0.03	0.11
Age	0.04^∗^	0.02	0.05^∗∗^	0.02	0.05^∗∗^	0.02	0.05^∗∗^	0.02	0.05^∗∗^	0.02
Education	0.10	0.12	0.11	0.11	0.14	0.11	0.12	0.11	0.14	0.11
Tenure	–0.02	0.02	–0.02	0.02	–0.03	0.02	–0.02	0.02	–0.03	0.02
Financial industry	0.24	0.17	0.26	0.17	0.24	0.17	0.22	0.12	0.22	0.17
Service industry	0.16	0.22	0.14	0.12	0.08	0.12	0.13	0.12	0.08	0.12
Independent variable										
Self-perceived status			0.16^∗∗^	0.06	0.09	0.06	0.12^∗^	0.06	0.08	0.06
Interactors:										
Cooperative behavior intention					0.07	0.06			0.07	0.06
Competitive behavior intention							–0.05	0.05	–0.03	0.06
Interactions										
Self-perceived status ^∗^ cooperative behavior intention					0.22^∗∗∗^	0.07			0.16^∗^	0.07
Self-perceived status ^∗^ competitive behavior intention							–0.20^∗∗^	0.07	−0.14^∗^	0.07
*R*^2^	0.06		0.09		0.14		0.13		0.16	
AR^2^	0.03		0.06		0.10		0.09		0.11	
*F*	1.98		2.88^∗∗^		3.57^∗∗∗^		3.40^∗∗∗^		3.33^∗∗∗^	

*^∗^*p* < 0.05, ^∗∗^*p* < 0.01, ^∗∗∗^*p* < 0.001 (two-tailed).*

## Discussion

In this study a theoretical model was developed to illustrate why and when self-perceived status promoted employees’ proactive helping behavior. The results revealed that self-perceived status was positively related to employees’ proactive behavior. Cooperative behavior intention increased the positive relationship between self-perceived status and employees’ proactive helping behavior, but competitive behavior intention decreased this positive relationship.

### Theoretical Contributions

This research makes several theoretical contributions. The most significant is that by using status competition theory as the foundation, a theoretical model was empirically tested that considers the potential influence of self-perceived status on the employee extra-role behavior of proactive helping. Previous research has mainly focused on the connection between self-perceived status and voice behavior (Janssen and Gao, 2015), after the construct of proactive helping behavior was first devised by Spitzmuller and Van Dyne (2013). General investigations of the relationship between helping behavior and individual status have been conducted; their results have indicated that people can elevate their social status through helping behavior (Flynn et al., 2006; Willer, 2009), and that seeking help from others may undermine individual status (Agneessens and Wittek, 2012). To the best of our knowledge, however, the relationship between self-perceived status and proactive helping behavior has not previously been explored or empirically tested. Therefore, the positive effect of self-perceived status on employee proactive helping behavior found in this study substantially extends the scope of employee behavioral outcomes, as motivated by their self-perceived status, to include an important but often neglected type of employee performance.

This study also contributes to the research on proactive helping behavior. Employee proactive helping behavior has been found to be positively promoted by leadership styles such as authentic leadership (Hirst et al., 2016) and transformational leadership (Zhu and Akhtar, 2014). Our study indicates the significant role of another unique and effective antecedent, self-perceived status, in determining employee proactive helping behavior. Based on the theory of the expected state, it has been suggested that the offer of proactive help to others depends greatly on the expected state of the helper (Nadler and Chernyak-Hai, 2014). However, our study indicates that employees who offer proactive help to their colleagues are motivated by their need for status competition and that those with higher self-perceived status have a higher willing to help, and the results consistent with the viewpoints of Brandts et al. (2015) and Willer (2009). Our study, therefore, helps to identify the antecedents of proactive helping behavior.

This study is also unique in identifying the contexts for the relationship between self-perceived status and employee proactive helping behavior. The moderators (i.e., cooperative behavior intention and competitive behavior intention) in this research are related to individuals’ status-contrasting strategy selection in the workplace (Chang et al., 2017). The increasing role of cooperative behavior intention in the association of self-perceived status with proactive helping behavior indicates that as cooperative behavior intention increases, high self-perceived status employees will increase their proactive helping behavior more than their low self-perceived status colleagues. However, the attenuating role of competitive behavior intention in the association of self-perceived status with proactive helping behavior indicates that as competitive behavior intention increases, high self-perceived status employees will decrease their proactive helping behavior more obviously than their low self-perceived status peers. These results show that in the development of proactive helping behavior, cooperative behavior intention and competitive behavior intention act as two completely reversed boundary conditions (promoter vs. inhibitor) of the influence of self-perceived status and proactive helping behavior. Therefore, the results further supported the suggestions of Chang et al. (2017). In addition, previous examinations of whether competition or cooperation is more productive have been inconclusive (Johnson, 2003; Chang et al., 2017), but the findings of this study suggest that cooperative behavior intention is more productive than competitive behavior intention.

Finally, our study also contributes to the literature on individual behavior intentions. Gould (2002) and Anderson et al. (2015) believed that an individual’s status-contrasting strategic choice depends greatly on their behavior orientation, and the relationship between different orientations and different competitive behaviors because of that different behavior orientations will focus on different resource stakes or approaches. From the team perspective, Chang et al. (2017) conducted an empirical test and found that whether the competition status of a group is established via cooperative or competitive behavior depends on the context. No studies have been found that empirically test the effects of cooperative versus competitive behavior intention on employee proactive helping at the individual level. Thus, following the suggestions of Gould (2002) and Chang et al. (2017), we analyzed and empirically tested behavior intention, and found that cooperative behavior intention acts as a promoter of employees’ proactive helping behavior. However, competitive behavior intention acts as an inhibitor that undermines employees’ proactive helping behavior, and thus supported the ideas of Gould (2002) and Anderson et al. (2015). This study has clearly distinguished the effect of cooperative versus competitive behavior and the application range has been extended to the field of proactive helping behavior.

### Practical Implications

The opposite moderating effects of cooperative versus competitive behavior intention indicate that self-perceived status may not necessarily positively motivate employees’ proactive helping behavior. The results show that cooperative behavior intention can strengthen the relationship between self-perceived status and proactive helping behavior, while competitive behavior intention can reduce the relationship between self-perceived status and proactive helping behavior. The findings indicate that organizations should guide their employees to increase their individual cooperative behavior intentions and thus improve their helping behavior. They also suggest that employees’ competitive behavior intentions should be discouraged, thus leading to higher proactive helping behavior. Therefore, organizations should create a work environment with a low level of competition (Fletcher and Nusbaum, 2010) and a high level of cooperation (Chatman and O’Reilly, 2004). Through such initiatives, an organization can motivate its employees to increase their individual proactive helping behavior, and thus contribute to achieving the organization’s goals.

### Limitations and Future Research

This study has several limitations. First, we measured employees’ proactive helping behavior through their supervisors. Although using data on employee behavior outcomes evaluated by supervisors is a normal practice in academia (Li et al., 2016), factors such as supervisor and subordinate Guanxi orientation may have affected the scoring, as the data were collected in China, which is a relationship-oriented country (Han et al., 2012). Therefore, future research could improve on our methodology by using peer evaluations of proactive helping behavior. Second, the mediating mechanisms between self-perceived status and proactive helping behavior were not analyzed and empirically tested in our study. Based on self-determination theory (Deci and Ryan, 1985), we suggest that role-breadth self-efficacy is most likely to be the mediator between the self-perceived status and proactive helping behavior. Third, the focus was only on the relationship between self-perceived status influence and proactive helping behavior; we did not distinguish the relationship between self-perceived status and proactive vs. reactive helping behavior. Lee et al. (2019) found that providing help without being asked (proactive helping behavior) differed from providing help when requested (reactive helping behavior). Therefore, this is also a direction warranting further research. Fourth, this research had not controlled the effect of employees’ hierarchical positions, this would be another limitation. An employee’s position in an organization might affect his/her self-perceived status (Anderson et al., 2012a). Therefore, future research needs to control hierarchical positions. Finally, although we had empirical test the moderated effects of individual behavior intentions (i.e., cooperative behavior intention and competitive behavior intention), we had not explored the moderated effects of individual status motivations. For instance, Loch et al. (2001) believed that there were two types of individual status motives: rational vs. emotional. And the rational motive guides status contrasting by resource stakes, but the emotional motive leads status contrasting by means. And Anderson et al. (2015) also suggested that it is worthwhile to distinguish the effects of the dominance-based from the prestige-based. Therefore, future research can deepen our understanding of the influence of self-perceived status on proactive helping behavior by considering those effects of individual status motives in the research model.

## Data Availability Statement

The raw data supporting the conclusions of this manuscript will be made available by the authors, without undue reservation, to any qualified researcher.

## Ethics Statement

The studies involving human participants were reviewed and approved by the Hubei University. The patients/participants provided their written informed consent to participate in this study.

## Author Contributions

CD developed the research model, analyzed the data, and co-drafted the manuscript. SL collected the data and co-drafted the manuscript. ZL edited the manuscript.

## Conflict of Interest

The authors declare that the research was conducted in the absence of any commercial or financial relationships that could be construed as a potential conflict of interest.
